# Genome-Wide Analysis of KNOX Transcription Factors and Expression Pattern of Dwarf-Related *KNOX* Genes in Pear

**DOI:** 10.3389/fpls.2022.806765

**Published:** 2022-01-28

**Authors:** Jianlong Liu, Chenxiao Zhang, Jingyue Han, Xiaoyun Fang, Hongpeng Xu, Chenglin Liang, Dingli Li, Yingjie Yang, Zhenhua Cui, Ran Wang, Jiankun Song

**Affiliations:** ^1^College of Horticulture, Qingdao Agricultural University, Qingdao, China; ^2^Haidu College, Qingdao Agricultural University, Laiyang, China

**Keywords:** pear, KNOX, genome-wide analysis, functional characterization, dwarf mechanism

## Abstract

KNOTTED1-like homeobox (KNOX) transcription factors (TFs) belonging to the homeobox TF family play important roles in plant growth, development, and responses to abiotic and biotic stress. However, little information is available on KNOX TF in pear (*Pyrus*). In this study, 19 PbKNOXs TFs were re-identified in pear (*Pyrus bretschneideri* Rehd.). Phylogenetic analysis revealed that the TFs were clustered into three groups with 10 conserved motifs, some of which were group- or subgroup-specific, implying that they are important for the functions of the KNOX in these clades. *PbKNM1* and *PbKNM2* are *KNM* (encodes a MEINOX domain but not a homeodomain) genes identified in pear for the first time. *KNOX* genes in *Pyrus* and *Malus* were closely related, and a collinear relationship among *PbKNOX* genes in *Pyrus* and *Malus* was observed. Analysis of the expression patterns of *PbKNOX* genes in different tissues, at various growth stages, and in response to abiotic and biotic stress revealed that *PbKNOXs* are involved in plant growth and development. Our comparative transcriptional analysis of dwarf mutant varieties revealed that genes belonging to class I are highly expressed compared with genes in other classes. Analysis of the expression of *PbKNOX* genes in the hybrid offspring of vigorous and dwarf varieties revealed that *PbKNOX* genes were highly expressed in the vigorous offspring and weakly expressed in the dwarf offspring. These findings provide new insight into the function of KNOX TFs in pear and will aid future studies of dwarf fruit trees.

## Introduction

Pear is one of the world’s most important fruits. In 2019, the world planting area of pear reached 1,379,387 ha, and the total output reached 23,919,075 tons. China’s output of pear accounts for 71.4% of the world’s output (FAO, 2021; http://www.fao.org/faostat/zh/#data/QCL/visualize). Labor-saving cultivation modes, such as dwarf close planting, have become important cultivation modes for fruit trees both within and outside of China for their various advantages, including early fruiting, high yield, high quality, ease of management, low costs, and high profit. However, the shortage of dwarf rootstocks or dwarf varieties has greatly impeded the development of the pear industry. The mechanism of dwarfism has become a major focus of research because of the development of biotechnology breeding.

Dwarfism is typically a result of reduced cell division and/or cell elongation. These processes are generally regulated by regulatory genes. Plant transcription factors (TFs) are important regulators that activate or inhibit the transcription of downstream genes during plant development and in response to environmental stimuli. Multiple TFs can play a role in dwarfism, including WRKY, BHLH, ARF, NAC, and homeobox TFs (Kato et al., 2010; Ou et al., 2015; Zhang et al., 2015). Previous studies have shown that there is an important relationship between internode development and shoot tip meristem and that homeobox family genes play an important role in shoot tip development. The homeobox is a conserved 60-amino acid motif called the homeodomain (HD), which can bind to specific DNA sequences. The HD has a three-helix structure and plays a role in development in all eukaryotic organisms. HD-containing proteins can be classified into six groups based on differences in the sequence, size, and location of the HD: homeodomain-leucine zipper (HD-Zip), Wuschel-related homeobox (WOX), plant homeodomain associated to a finger domain (PHD-finger), bell domain (BELL), knotted related homeobox (KNOX), and zinc finger-homeodomain (ZF-HD; Moens and Selleri, 2006; Ariel et al., 2010).

KNOX family members have been shown to be involved in plant dwarfism and internode development. The first *KNOTTED1* gene to be identified in plants was *KNOTTED1* (*kn1*) in maize (Vollbrecht et al., 1991). Following this discovery, several studies of KNOTTED1 proteins have been carried out in model and non-model plants, named KNOTTED1-like. The functions of KNOX proteins have been studied extensively in *Arabidopsis*, rice, and cotton (Bhattacharjee et al., 2016; Yang et al., 2017; Wang et al., 2019). Based on sequence similarity, intron position, expression patterns, and phylogenetic analysis, the *KNOX* genes can be divided into two subclasses: class I and class II (Furumizu et al., 2015). The functions of class I *KNOX* genes have been extensively studied. In *Arabidopsis*, there are four class I genes: *SHOOTMERISTMELESS* (*STM*), *KNAT1(BP)*, *KNAT2*, and *KNAT6*. The gene *STM* is essential for the formation and maintenance of the shoot apical meristem (SAM; Aguilar-Martinez et al., 2015). KNOX1 proteins form heterodimers with other HDs (e.g., BEL-like HD) in the TALE superclass and regulate the activity of downstream genes with different combinations of KNOX/BLH TFs (Arnaud and Pautot, 2014). BELL1-like homeobox (BLH) TFs are known to heterodimerize with KNOTTED1-like homeobox TFs and play important roles in shoot meristem maintenance, maintain intercalary meristems that accumulate KN1, and promote internode elongation (Tsuda et al., 2017). Other class I proteins can regulate flower patterning, promote the outgrowth of primordia, create leaf tips, and create the basipetal streams of auxin from the tip through the internal tissue to induce the differentiation of the vascular strand (Thomas et al., 2006; Wenzel et al., 2007; Li et al., 2012a).

Among class II genes, *MdKNOX19* in apple is a positive regulator of *ABI5* expression, and the conserved module MdKNOX19-MdABI5-ABA may contribute to organ development. However, functional studies examining class II genes are fewer than those examining class I genes (Jia et al., 2021). Class I and class II genes perform non-redundant functions to control the development of all the aboveground organs of plants (Furumizu et al., 2015). With the continually increasing amount of plant genome data published, genome-wide identification and analysis have become effective methods for rapidly predicting gene functions from a large family of genes. Several gene families in plants have been studied. Eighteen *PbKNOXs* family genes were identified in pear. Based on the phylogenetic tree and chromosomal localization, the 18 *PbKNOX* genes were divided into five subfamilies [SHOOT MERISTEMLESS (STM)-like, BREVIPEDICELLUS (BP)-like, KNOTTED ARABIDOPSIS THALIANA 2/6 (KNAT2/6)-like, KNAT7-like, and KNAT3-5-like] and were distributed among 10 chromosomes (Cheng et al., 2019). With the improvement of genomic data, the bioinformatics of these PbKNOXs have changed, such as the characteristics of protein, sequence information, and chromosome location. Therefore, in this study, we re-identified the relevant information to improve the accuracy of sequence information and bioinformatics information of related proteins.

According to previous studies, we speculate that *KNOXs* family genes are important dwarfing-related genes. Dwarf negative regulatory genes are important for genetic engineering breeding by gene editing. However, dwarfism is often accompanied by weak plant growth and survival challenges for plants. “Nain Vert,” a French pear cultivar with short internodes and stature, originated from a chance seedling (Bagnara and Rivalta, 1989). This germplasm resource can be used in crossbreeding to create new dwarf scion cultivars with high quality. We introduced seeds of “Nain Vert” from the East Malling Research Station, West Malling, United Kingdom, in 1991. Several seedlings were then raised, and one dwarf seedling showing similar features to “Nain Vert” was identified and named “Aihuali.” “Chili,” which belongs to *Pyrus bretschneideri* Rehd., is famous for its fruit quality and is mainly cultivated in Shandong Province, China. It has higher photosynthetic capacity, short internodes, and thick branches; it is thus an excellent material for studying the mechanism underlying dwarfism.

Here, we re-conducted a genome-wide analysis of Chinese white pear (*Pyrus bretschneideri* Rehd.) *PbKNOXs* investigated their potential functions in plant development and responses to stress. We used dwarf pear materials to identify the expression pattern of *PbKNOXs* in dwarfism.

## Materials and Methods

### Database Search and Sequence Retrieval

The complete genome assembly of pear (*Pyrus bretschneideri* Rehd.) along with the complete proteome sequence file was downloaded from the Genome Database for Rosaceae.^1^ Conserved amino acid sequences were used as a query to identify PbKNOXs TFs in the *Pyrus* proteome sequence file using CLC Sequence Viewer v7.6.1 (Jin et al., 2017).

Putative PbKNOX TFs were confirmed by BLASTP searches of the NCBI database. This database was also used to obtain gene accession numbers, chromosome number, genomic information, and the protein size of KNOX TFs. The nucleotide sequences of all identified *PbKNOX* were also retrieved from NCBI. The ExPASY online tool^2^ was used to calculate molecular weight, pI, and GRAVY of all PbKNOX proteins. WoLF PSORT^3^ was used to predict the subcellular localization of PbKNOX proteins.

### Chromosomal Mapping, Intron/Exon Distribution, Conserved Domain, and *cis*-Elements Analysis

The NCBI database was used to record the positions of the identified *PbKNOX* genes on chromosomes, and Map Chart v2.32 was used to draw a map of chromosomal locations of *PbKNOX* genes to scale (Voorrips, 2002). Gene Structure Display Server (v2.0 http://gsds.cbi.pku.edu.cn/) was used to draw and visualize the intron/exon organization of *PbKNOX* genes (Hu et al., 2014). The whole genome sequence and the coding sequences of all *PbKNOX* genes were used to construct the gene structure map containing introns. In order to identify domains conserved among all PbKNOXs, their protein sequences were analyzed with MEME v5.0.3 (Bailey et al., 2015) using default parameters, except that the minimum number of motif sites was set to 10. The 2,000-bp sequence upstream from each *PbKNOX* initiation codon was analyzed, and the online tool PlantCARE^4^ was used for the prediction of *cis*-elements (Lescot et al., 2002).

### Phylogenetic Comparison of Homoebox and KNOX Proteins in *Pyrus bretschneideri*, *Malus domestica*, and *Arabidopsis thaliana*

A phylogenetic tree was constructed using the protein sequences of putative Homeobox TFs from *Pyrus bretschneideri*. Another phylogenetic tree was constructed using the protein sequences of putative KNOX TFs from *Pyrus bretschneideri* and *Malus domestica*, with *Arabidopsis thaliana* sequences used as a reference (Supplementary Table S1). Multiple sequence alignment of the proteins was performed using ClustalW v1.83. The tree was used to infer the evolutionary history and functions of PbKNOX TFs. Their coding sequences were used for pairwise alignment with the built-in ClustalW and PAM protein weight matrix of MEGA7 (Kumar et al., 2016). The resultant alignments were analyzed for DNA sequence polymorphisms and DnaSP v5.10.01 (Librado and Rozas, 2009) was used to compute the synonymous substitution rate (Ks) and nonsynonymous substitution rate (Ka). Ks/Ka was also calculated to evaluate codon selection during evolution.

### Expression Patterns of *PbKNOXs* in Various Tissues of Pear at Different Developmental Stages

The expression patterns of *PbKNOX* genes in various tissues at different developmental stages were obtained from the pear gene expression atlas. Bud was collected from flower bud differentiation stage. Leaf, petal, sepal, oval, and stem were collected from full flowering stage. Stem was the stem tissue of annual branches, and fruit was collected from commercial maturity stage. The raw sequence reads from seven pear tissues were analyzed using the NCBI web browser (https://www.ncbi.nlm.nih.gov/bioproject/; accession no. PRJNA498777; Li et al., 2019a). The expression patterns of *PbKNOX* genes at three different stages of ovary development and under three treatments [unpollinated (control), hand-pollinated, and unpollinated overy with GA treatment] were analyzed (Liu et al., 2018a, 2021). For the analysis of expression patterns of *PbKNOX* genes in leaf samples at different developmental stages of pear, leaves were collected at 30, 45, 60, 75, and 90 DAB; the RNA sequencing (RNA-seq) data were downloaded from the Sequence Read Archive (SRA; accession nos. SRR10997902–SRR10997912; Zhao et al., 2020). Finally, the expression profiles of *PbKNOX* genes based on RNA-seq data from four pear varieties at seven fruit development stages were downloaded from the SRA (accession no. SRP070620). The following fruit development stages were analyzed as: fruit setting (period 1), physiologic fruit dropping (period 2), rapid fruit enlargement (period 3), 1 month after fruit enlargement (period 4), pre-maturity (period 5), and maturity (period 6); additionally, one fruit senescence stage after harvest (period 7) was included in the analysis (Zhang et al., 2016).

### Expression Patterns of *PbKNOXs* Under Different Stress Conditions

Expression patterns of *PbKNOXs* in response to abiotic and biotic stress were based on microarray data downloaded from the SRA (series matrix accession nos. SRP051914 and SRP148620; Yang et al., 2015; Li et al., 2016) and data from the present study.

#### Salt Stress

Two varieties of pear, “QAUP-1” (*Pyrus ussuriensis* Maxim.) and “Qingzhen D1” (*Pyrus communis* L. × *Pyrus bretschneideri* Rehd.), were used. Salt stress treatment was applied as: half-strength nutrient solution and 100 mmol/L NaCl. Leaf and root samples were collected from the plants 0, 12, and 24 h after treatment along with control samples. Each treatment contained three replicates of 50 plants (Liu et al., 2021).

#### Drought Stress

*Pyrus betulaefolia* plants used in this experiment were 3 months old.

Seedlings were transferred to clean filter paper (90 mm × 90 mm) and allowed to dry for 0, 1, 3, and 6 h at 26°C, followed by recovery in water at 26°C for 24 h. Each treatment contained three biological repeats (Li et al., 2016; Liu et al., 2021).

#### Cork Spots

The superior pear line “1–43” is the hybrid from “Xinli No. 7” (*Pyrus bretschneideri* Rehd.) × “Zhongxiang Pear” (*Pyrus bretschneideri* Rehd.). Its fruit showed cork spots at 180 days after anthesis. We took normal pear flesh without disease, pear flesh with moderate disease, and pear flesh with serious cork spots 180 days after flowering as materials, including three biological repeats (Liu et al., 2021).

#### Pear Black Spot Disease

The spore suspension was sprayed into detached young leaves of “Jinjing” and “Hongfen” pear varieties with a glass atomizer. Control leaves were sprayed with distilled water. Samples H-CK, H-P, J-CK, and J-P were used in the experimental design and data analysis. All of the samples were tested in triplicate, and the experiments were performed on three biological replicates (Yang et al., 2015; Liu et al., 2021).

### Expression Patterns of *PbKNOXs* in Pear Varieties With Different Growth Potential

The experiment was carried out in the Jiaozhou Experiment and Demonstration Station of Qingdao Agricultural University (36.44°N, 120.09°E), which features a warm temperate monsoon climate. The branches of dwarfing pear “601D” were treated with Co-r (60; 5,000 rad) radiation and then grafted on the rootstocks of one-year-old *Pyrus betulifolia* Bunge. One vigorous mutant was observed and referred to as “601V.” “601D” and “601V” were then grafted on the five-year-old rootstocks of *Pyrus betulifolia* Bunge. “Laiyang chili” pear and dwarf pear were used for hybridization. There were two phenotypes in the hybrid offspring population: non-dwarf varying in height and dwarf. Fifteen individual trees of arboreal and dwarf progenies were selected for the expression pattern analysis of *PbKNOX* genes. The samples were collected from the shoot tips of three-year-old branches. All samples were immediately placed into liquid nitrogen and then stored at −80°C until further use.

### Plant Growth Conditions and Quantitative Real-Time PCR Analysis

Total RNA of pear peel samples at the three different stages was extracted using the RNAprep Pure Plant Plus Kit (Tiangen, Beijing, China) and treated with DNase I (RNase-free; Takara Bio, Dalian, China) to eliminate residual contaminating genomic DNA. For qRT-PCR, 1.5 μg total RNA was used to synthesize first-strand cDNA with the PrimeScript II First-Strand cDNA Synthesis kit (Takara Bio) according to the manufacturer’s instructions. qRT-PCR amplification was carried out as follows: 95°C for 5 min, 45 cycles at 95°C for 15 s, 60°C for 30 s, and 72°C for 30 s using the Roche 480 real-time PCR system (Basil, Switzerland) in standard mode with the FastStart Essential DNA Green Master kit. All reactions were performed in triplicate at a volume of 20 μl, containing 2 μl of 10-fold diluted cDNA. The Pyrus ACTIN gene was used as the reference to normalize the qRT-PCR data, and relative gene expression levels were determined *via* the 2^−ΔΔCT^ method (Livak and Schmittgen, 2001). All experiments were performed with three biological replicates and all primer sequences are shown in Supplementary Table S2.

### Statistical Analysis

Statistical analyses were conducted using SPSS 23.0 (SPSS, Chicago, IL, United States). Values are represented as the mean ± SD of three independent biological replicates. Data were analyzed with Duncan’s test, and *p* ≤ 0.05 was considered significant.

## Results

### Identification and Phylogenetic Analysis of PbKNOX TFs in Pear

**Figure 1:**
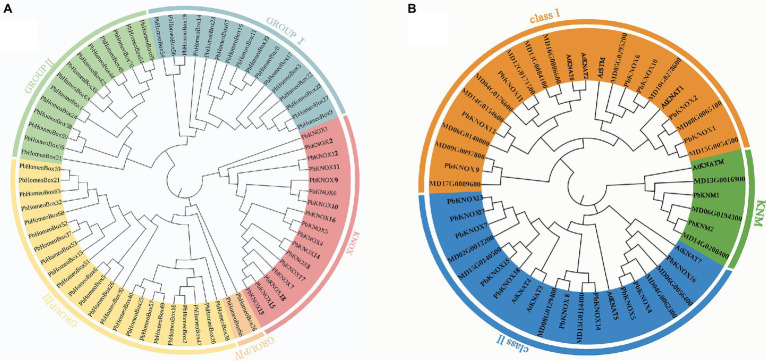
Phylogenetic tree constructed with the neighbor-joining method using homeobox TF domains in *Pyrus bretschneideri* **(A)** and KNOX TF domains in *Pyrus bretschneideri*, *Malus domestica*, and *Arabidopsis* **(B)**. The tree was divided into three groups (class I, class II, and KNM).

Putative pear PbKNOX sequences were identified by searching the Rosaceae genome using the Basic Local Alignment Search Tool for protein sequences (BLASTP). A total of 19 KNOX protein sequences were screened based on domain and named according to Cheng’s research (Table 1; Cheng et al., 2019). However, the PbKNOXs protein sequence has been modified in the latest reference genome. In previous studies, PbKNOX3 was not located on the chromosome, and no similar sequence was found in the latest version, which was deleted (Supplementary Table S1). Two *PbKNM* genes were found according to the conserved domain. All of the identified family members contained the KNOX I and/or KNOX II domains. The polypeptide lengths of the predicted pear PbKNOXs ranged from 141 to 450 amino acids. The predicted subcellular localization was the nucleus for all *PbKNOXs*. The molecular weight, isoelectric point (pI), and grand average of hydropathicity (GRAVY) are summarized in Table 1. We examined the phylogenetic relationships among pear homeobox and *PbKNOX* genes by generating a phylogenetic tree using the neighbor-joining method. A total of 72 homeobox genes were identified in pear, and the family was divided into five groups. Among them, 17 genes in KNOX group are *PbKNOX* genes. Therefore, we analyzed the evolutionary relationships among these 19 genes with 21 *Malus domestica* and eight *Arabidopsis KNOX* genes (Figure 1). The family was divided into three groups (Figure 1). *PbKNOX6*, *PbKNOX2*, *PbKNOX9*, *PbKNOX10*, *PbKNOX11*, *PbKNOX12*, and *PbKNOX1* were more closely related to *STM*, *KNAT1*, *KNAT2*, and *KNAT6*, which belong to the class I family. *KNATM* and three *KNM*s of apple were closely related to *PbKNM1* and *PbKNM2*. The remaining *PbKNOXs* are homologous to class II genes.

**Table 1:** General information on *Pyrus bretschneideri KNOX* genes.

Gene name	Gene ID number^1^	Chromosome	Start site	Termination site	Amino acid residues	MW (Da)	pI	Hydrophilicity	Subcellular localization^2^
*PbKNM1*	rna10018	chr6	20,008,418	20,010,497	141	15638.53	4.76	−0.609	nuclear
*PbKNM2*	rna14130	chr14	18,530,146	18,531,928	141	15768.78	4.72	−0.567	nuclear
*PbKNOX1*	rna1583	chr15	4,076,074	4,080,842	397	44989.74	6.03	−0.988	nuclear
*PbKNOX2*	rna38189	chr8	14,101,277	14,105,532	399	45251.96	6.06	−1.009	nuclear
*PbKNOX4*	rna32565	chr4	16,174,737	16,181,204	288	32834.04	6.31	−0.722	nuclear
*PbKNOX5*	rna32562	chr4	16,229,314	16,235,782	288	32834.04	6.31	−0.722	nuclear
*PbKNOX6*	rna132	chr5	26,271,358	26,276,271	381	42495.34	6.28	−0.738	nuclear
*PbKNOX7*	rna43007	chr2	1,877,460	1,880,440	371	41999.67	5.42	−0.764	nuclear
*PbKNOX8*	rna16378	chr8	8,400,312	8,404,344	450	50031.37	5.86	−0.752	nuclear
*PbKNOX9*	rna19228	chr9	15,005,982	15,008,793	333	37605.38	6.32	−0.652	nuclear
*PbKNOX10*	rna11518	chr10	24,786,052	24,791,355	391	43845.96	6.32	−0.731	nuclear
*PbKNOX11*	rna27810	chr12	17,373,069	17,376,582	320	35876.97	5.09	−0.667	nuclear
*PbKNOX12*	rna17030	chr14	19,211,788	19,219,322	348	38949.59	5.21	−0.629	nuclear
*PbKNOX13*	rna20287	chr2	1,038,583	1,041,283	359	40697.29	5.48	−0.738	nuclear
*PbKNOX14*	rna9835	chr15	9,055,024	9,058,734	448	49885.18	5.89	−0.774	nuclear
*PbKNOX15*	rna12545	chr15	10,387,277	10,390,009	358	40532.99	5.29	−0.768	nuclear
*PbKNOX16*	rna16886	chr6	6,976,865	6,982,613	288	32847.03	6.31	−0.739	nuclear
*PbKNOX17*	rna43058	chr2	2,101,752	2,104,735	371	41999.67	5.42	−0.764	nuclear
*PbKNOX18*	rna39538	NW_008988522.1	240,485	243,159	369	41692.23	5.29	−0.792	nuclear

MW, molecular weight; pI, theoretical isoelectric point.

1 From *Pyrus bretschneideri* genome sequence consortium database.

2 Predicted using WoLFPSORT (http://www.genscript.com/psort/wolf_psort.html).

### Chromosomal Distribution and Orthologous Relationships of *PbKNOX* Genes in *Pyrus bretschneideri*, *Malus domestica*, and *Arabidopsis thaliana*

**Figure 2:**
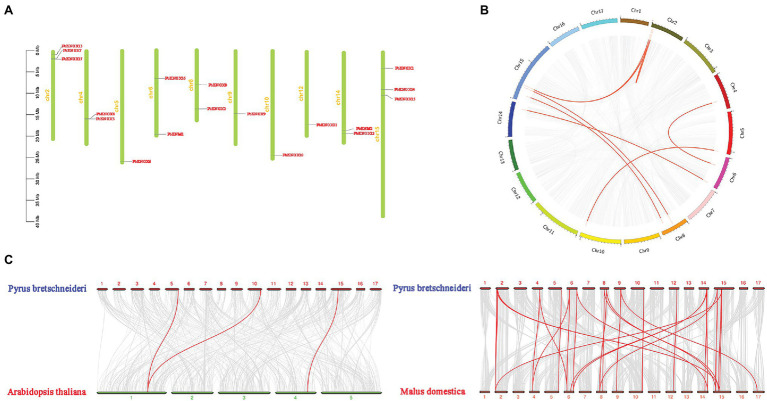
Chromosomal distribution of *PbKNOX* genes drawn using MapChart v2.2. The scale represents chromosome length: 0–40 Mb **(A)**. Collinearity of *KNOX*s **(B)**. Distribution of segmentally duplicated *PbKNOX* genes on *Pyrus bretschneideri* chromosomes. Gray lines indicate collinear blocks in the whole *Pyrus bretschneideri* genome, and red lines indicate duplicated *PbKNOX* gene pairs. **(C)** Collinearity of *Pyrus bretschneideri*, *Arabidopsis*, and *Malus domestica KNOX* genes. Red lines indicate syntenic gene pairs.

Physical mapping of *PbKNOXs* on the 17 chromosomes of *P. bretschneideri* revealed that the distribution of *PbKNOXs* was uneven (Figure 2); the genes were distributed on 10 chromosomes, with one or two genes per chromosome. The scaffold of *PbKNOX18* genome database is not attached to the chromosome, which may be a problem of gene assembly. The exact position (in bp) of each *PbKNOX* is shown in Table 1. Two *PbKNOX* genes were tandem repeats, and 12 (six pairs) were segmentally duplicated (Figure 2B). The Ka/Ks ratios of the gene pairs displaying segmental duplication were less than 1, indicating that they might have undergone purification selection (Supplementary Table S4). To establish the orthologous relationships of PbKNOXs, we compared the physical location of PbKNOXs in the genomes of *Pyrus bretschneideri*, *Malus domestica*, and *Arabidopsis thaliana* (Figure 2C). PbKNOX6, PbKNOX10, and PbKNOX1 were closely related to KNOX in *Arabidopsis*. There was a collinear relationship among KNOX in *Malus* and *Pyrus*.

### Cis-Elements, Conserved Motifs, and Gene Structure of PbKNOX TFs

A total of 19 *PbKNOX* promoter regions (2,000 bp) were analyzed. There was a large number of *cis*-acting elements in the promoter region, which mainly included hormone signals and stress regulatory networks, such as the GARE-motif, P-box (related to gibberellins), CGTCA-motif, and TGACG-motif (related to JA; Supplementary Table S3).

**Figure 3:**
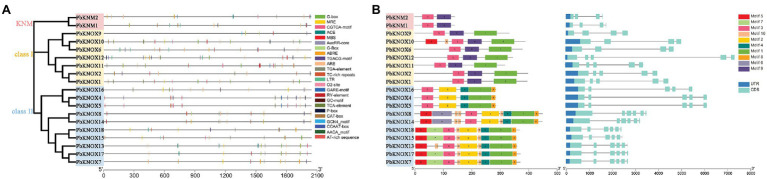
A list of motifs detected in the promoter regions of *PbKNOX* genes **(A)**. Schematic representation of protein and gene structures of *Pyrus bretschneideri PbKNOX* genes **(B)**. Motifs 1–10 identified using the MEME search tool are marked on the protein sequences in each clade (1–3). The length and order of each motif correspond to the actual length and position of each motif in the protein sequences, respectively. The coding sequence and untranslated regions are represented by filled dark blue and orange boxes, respectively. Evolutionary analysis shows the homology of PbKNOXs in pear.

The conserved motifs of PbKNOXs were analyzed using Multiple Em for Motif Elicitation (MEME). Ten conserved motifs were predicted in PbKNOXs (Figure 3; Supplementary Figure S1); their sizes ranged from 11 to 80 amino acids. Motif 3 was present in all PbKNOXs; motif 2 was detected in class II members; motif 1 was present in class I and class II group members; motif 9 was present in KNM and class I group members; the other motifs were observed in class II members; and motif 5 and motif 10 were detected in PbKNOX10. Most *PbKNOX* genes had 4 ~ 7 exons and 3 ~ 6 introns (Figure 3).

### Expression Pattern of *PbKNOX* Genes During Pear Development

**Figure 4:**
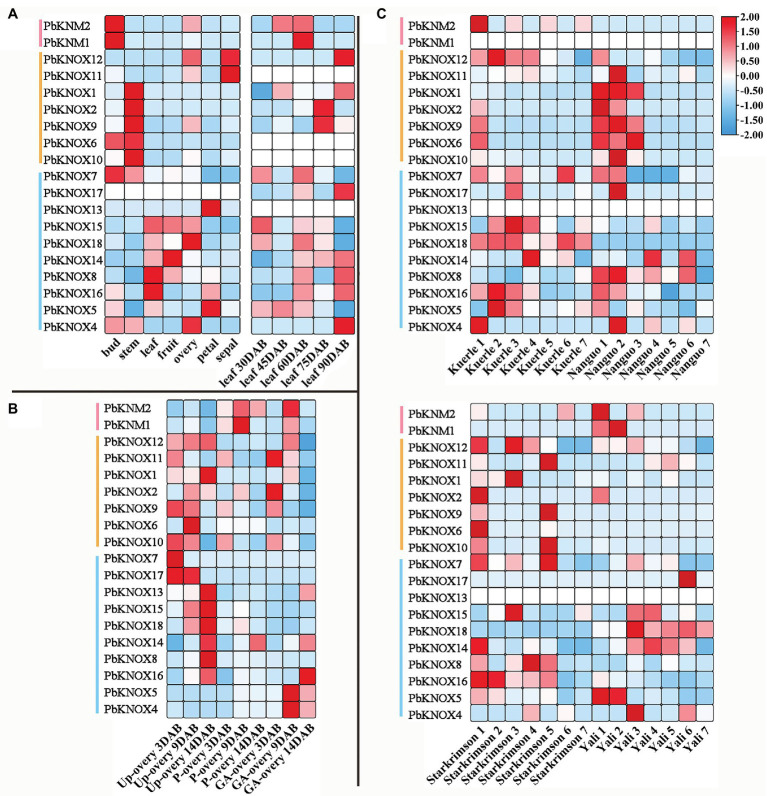
Expression profiles of *PbKNOX*s in pear. **(A,B)** Expression profile of *PbKNOX*s in leaf **(A)** and ovary at different development stages **(B)** of *Pyrus bretschneideri*. **(C)** Expression profile of *PbKNOX*s at different fruit development stages in various *Pyrus bretschneideri* varieties. P-ovary, pollinated ovary; Up-ovary, unpollinated ovary.

We analyzed the spatiotemporal expression patterns of *PbKNOX* genes in seven different tissues and at different developmental stages using publicly available gene expression data. Of the examined *PbKNOX* genes, one was not expressed in any of the tissue types; most of the genes were expressed in all seven tissue types, and their expression was constitutive (fragments per kilobase of transcript per million mapped reads (FPKM) > 10). Among class I genes, *PbKNOX6*, *PbKNOX2*, *PbKNOX9*, *PbKNOX10*, and *PbKNOX1* were highly expressed in buds and stems, especially stems (Figure 4). During leaf development, the expression of *PbKNOX5* decreased gradually with leaf development time in class II, and the expression of *PbKNOX16* and *PbKNOX14* increased gradually with leaf development time. However, the expression of genes was low in class I and KNM group, and there was no significant difference in the expression of these genes between these two groups (Figure 4A). During ovary development, the expression of genes in class I and class II was significantly different. In class I, the expression of *PbKNOX11/9/10* became downregulated as development progressed, and in class II, the expression of most genes became upregulated as development progressed (*PbKNOX13/15/18/14/8/16*; Figure 4B). *PbKNOXs* are involved in fruit development, and *PbKNOX14/8/12* were highly expressed (fragments per kilobase of transcript per million mapped reads (FPKM) > 10) in four pear varieties.

### Expression of *PbKNOX* Genes in Response to Abiotic and Biotic Stress

**Figure 5:**
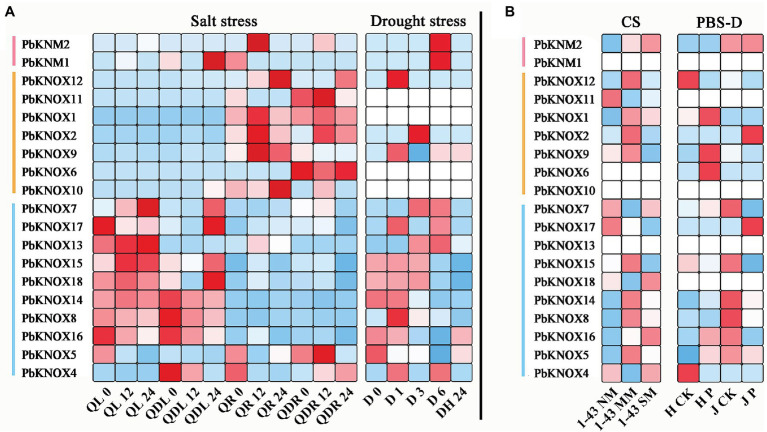
Expression profiles of *PbKNOX* genes under abiotic and biotic stress. **(A)** Abiotic stress included salt and drought stress. **(B)** Biotic stress included cork spot and pear black spot disease. CS, cork spot; MM, moderate disease mesocarp; NM, normal mesocarp; PBS-D, pear black spot disease; QL, “Qaup-1” leaf, QR, “Qaup-1” root; QZL, “Qingzhen D1” leaf; QZR, “Qingzhen D1” root; and SM, severe disease mesocarp.

To determine whether *PbKNOXs* expression is affected by different types of stress, we analyzed the expression patterns of genes following exposure to abiotic and biotic stress (Figure 5). Under salt stress, the expression of class I and class II group genes significantly differed among tissues. Most genes in class II group were highly responsive to salt stress in leaves, whereas genes in class I group were highly expressed in roots. Comparison of expression under drought stress and black spot infection revealed that the expression of class II group genes was high under different types of stress. The expression of class I and KNM group genes was lower across all treatments. The expression of *PbKNOX7/13* increased under drought stress and decreased after plants were covered with water, whereas the expression of *PbKNOX5/16* was inhibited under drought stress and increased after plants were covered with water.

### Expression of *PbKNOX* Genes in Dwarf Plants

**Figure 6:**
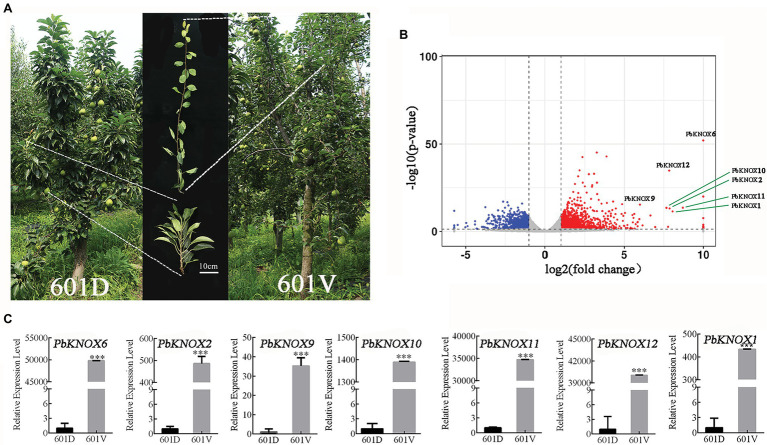
Expression profile of *PbKNOX*s in “601D” and the new vigorous mutant “601V.” **(A)** Photographs of “601D” and “601V.” **(B)** Volcano map of differentially expressed genes in the “601D” and “601V” transcriptome. **(C)** Expression profile of *PbKNOX*s in “601D” and “601V” pear. ***p < 0.001 (Student’s *t*-test). The expression levels of genes in “601D” pear under control conditions were normalized to 1.0.

We used the dwarf pear variety “601D” and its vigorous mutant “601V” to study the role of PbKNOXs in dwarf plants. The internodes of “601D” were shorter than those of “601V”; the tree body was dwarf, the tree was sturdy, and the branches were significantly higher than those of “601V” (Figure 6A). A total of 2,741 differentially expressed genes were identified, including 1,636 upregulated and 1,105 downregulated genes. PbKNOXs in class I were all differentially expressed (log2fold change >5; Figure 6B). The expression of *Pb*KNOX genes was much higher in “601V” than in “601D” (Figure 6C).

**Figure 7:**
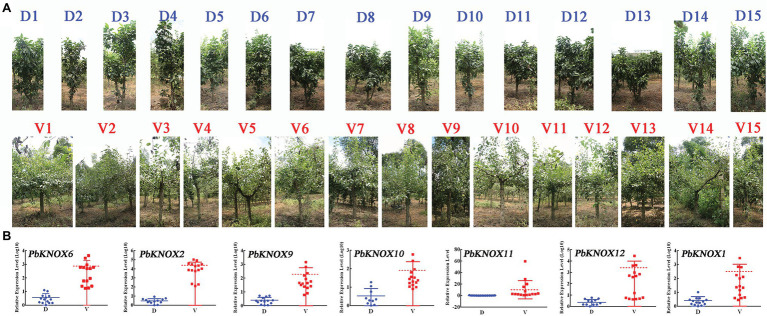
Expression profile of *PbKNOX*s of hybrid progeny. **(A)** Representative types of 15 dwarf and 15 vigorous pear trees. **(B)** Expression pattern of *PbKNOX* class I genes in offspring.

We used the hybrid progeny population of “Laiyang Chili” pear and dwarf pear to screen 30 plants: 15 non-dwarf plants and 15 dwarf plants. The dwarf population had the dwarf phenotype, which is characterized by short internodes and thick branches. The expression of *Pb*KNOX genes in class I group was significantly higher in vigorous plants than in dwarf plants (Figure 7).

## Discussion

Homeobox genes, including *HD-Zip*, *WOX*, *PHD-finger*, *BELL*, *KNOX*, and *ZF-HD* genes, have been studied in other species. These genes are involved in early leaf development and lateral branch growth. They also respond to different types of stress (e.g., aluminum stress and drought stress) and play a role in the signal transduction of multiple hormones (Nakata et al., 2012; Alvarez et al., 2015; Lim et al., 2018; Li et al., 2019b; Feng et al., 2020). Previous studies have conducted bioinformatics analysis on the *PbKNOX* gene family, and the research shows that there are 18 *PbKNOX* genes in pear. In our study, we named it according to sequence, chromosome location, and previous naming (Cheng et al., 2019). However, *PbKNOX3* is not displayed in the current study, because there is no chromosome location and there is no consistent sequence in sequence alignment. Meanwhile, two *PbKNM* genes were identified. Compared with previous studies, the sequence information of nine genes was corrected, of which seven had amino acid deletion. Due to incomplete chromosome assembly, the length of some chromosomes is not enough, and gene chromosome mapping is inaccurate. So we used the latest chromosome information to relocate all PbKNOXs. In this study, a total of 72 homeobox proteins were identified in *Pyrus bretschneideri*. There were 17 proteins in KNOX group. Evolutionary analysis of the homologs of pear PbKNOXs in *Arabidopsis* and *Malus* was conducted. *PbKNOX6/2/9/10/11/12/1* are closely related to *STM*, *KNAT1*, *KNAT2*, and *KNAT6* in *Arabidopsis*, indicating that these are class I genes. We identified two PbKNM paralogs in *Pyrus* and three paralogs in *malus*; PbKNM1 and PbKNM2 have high homology with MdKNM in apple. Conservative domain analysis of these two proteins revealed the loss of the homeobox domain. Previous studies have shown that KNATM family KNOX proteins (i.e., proteins with only KNOX1 and KNOX2 domains) occur exclusively in eudicots, and some species contain more than one homolog of the KNM protein (Gao et al., 2015). *PbKNM1* and *PbKNM2* are *KNM* genes identified in pear for the first time in this study.

The number of *PbKNOX* genes in pear (19) is greater than the number of *PbKNOX* genes in *Arabidopsis* (8); this might stem from gene duplication in plants, which is considered a fundamental driver of the evolution of genomes (Kong et al., 2007), as it provides raw material for new genes that can then lead to the emergence of new functions. Segmental duplication, tandem duplication, and transposition events, such as retro- and replicative transposition (Moore and Purugganan, 2003), are the three main mechanisms of gene evolution; the first two are thought to underlie gene family expansion in plants (Cannon et al., 2004). Tandem duplications can be identified based on the presence of multiple members of a single gene family within the same or in neighboring intergenic regions (Ramamoorthy et al., 2008). Among the 19 *PbKNOX* genes in pear, there were one groups of tandem repeats (*PbKNOX4* and *PbKNOX5*). We also detected six segmental duplication pairs involving 12 *PbKNOX* genes, suggesting that segmental duplication was the main driver of the expansion of *PbKNOX* genes in *Pyrus*. We also found that tandem repeats and segmentally duplicated pairs were concentrated in class II subfamily, which might indicate that the genes in the class I family are more conservative. Variation in the class II family contributes to the increase in the number of *PbKNOX* genes in pears. A collinear relationship of *PbKNOX6* and *PbKNOX10* with *STM*, *KNAT2*, *KNAT4*, and *KNAT5* in *Arabidopsis* was detected. A total of 17 *PbKNOX* genes in pear were collinear with *KNOX* genes in *Malus*. Syntenic analysis of *KNOX* genes in *Pyrus*, *Arabidopsis*, and *Malus* showed that *PbKNOX* genes had higher homology with *KNOX* genes in *Malus* and lower homology with those in *Arabidopsis*.

In plants, gene expression is regulated by various physical and chemical factors. Regulation of expression at the transcriptional level is the most important mode of gene expression regulation. *Cis*-acting elements in the promoter, terminator, and UTR sequences play an important role in regulating expression. Several *cis*-elements responsive to plant growth regulation, auxin, GA, SA, biotic stress, and abiotic stress are present in the promoter of *PbKNOX* genes. This indicates that *PbKNOX* genes are induced by different factors, which is consistent with the observed expression patterns related to growth, development, and stress. Conserved domains or amino acid motifs in TFs are frequently involved in DNA binding, nuclear localization, protein–protein interactions, and transcriptional activity (Du et al., 2013), and TFs with similar domains or motifs likely have similar functions. *KNM* is a subfamily gene with the least conserved domain. The expression of *PbKNM1* and *PbKNM2* was generally low. Motif 2 and motif 4 conserved domains in class II group are unique, and motif 9 is unique in class I group. The distribution of these motifs may account for the variable responses of *PbKNOX*s to different conditions.

Based on the above analysis, we used transcriptome data to analyze the expression patterns of 19 *PbKNOX* genes in the process of growth and development and under biotic and abiotic stress to explore the possible functions of PbKNOXs. Most *PbKNOX* genes identified in our study are involved in these processes. Moreover, *PbKNOX* genes show significant tissue-specific expression, in which genes in class I group are highly expressed in growth point areas, such as the stem and bud, which indicates that class I genes play an important role in the function of the meristem and growth and development. Class I group genes have more cis-elements of P-box and CAT-box, which may have an important relationship with its functions of regulating growth and promoting meristem development. In other species, class I group genes have been shown to promote the development of lateral branches and internodes (Aguilar-Martinez et al., 2015; Tsuda et al., 2017). In class II group, the expression of *PbKNOX5* decreased gradually with leaf development time, whereas the expression of *PbKNOX14* and *PbKNOX16* increased gradually with leaf development time. Meanwhile, *PbKNOXs* is also highly expressed during ovary development and fruit development, indicating that it plays an important role in these processes.

Under salt stress treatment, the expression of most genes in class II group was highly responsive in leaves, whereas class I group genes were highly expressed in roots. This indicates that *PbKNOX* genes in class I and class II differ in their functions, especially in growth and development and the response to salt stress. This difference in expression might be related to differences in their conserved domains. Some members of the family also showed responses to drought stress, such as the increased expression of *PbKNOX7/13* under drought stress and after plants were covered with water. The expression of *PbKNOX5/16* was inhibited under drought stress and increased after plants were covered with water. *PbKNOX* genes in pear have important regulatory functions in growth and development and responses to stress.

*PbKNOX*s play an important role in the regulation of dwarf plants. We examined the specific expression pattern of *PbKNOX* genes in internode development in the pear varieties “601D” and “601V.” “601D” had an obvious dwarf phenotype compared with “601V,” and its internodes were significantly shorter than those of “601V.” Transcriptomic data showed that class I genes were significantly overexpressed in “601V,” and their log2fold change was 5-fold higher compared with “601D.” Class I genes play an important role in SAM. Loss of STM function prevents the formation of SAM and thus the continuous generation of lateral organs; this inhibition of growth leads to dwarf but strong plants (Liu et al., 2018b), which is consistent with our findings. In order to further verify the expression pattern of class I gene in dwarf plants, we used the dwarfing hybrid population to screen 30 seedlings showing significant variation in height in the offspring population, including 15 vigorous and 15 dwarf trees. The expression of *PbKNOX* genes was higher in offspring plants than in dwarf plants. This indicates that class I genes contribute to dwarfism in plants. Previous studies have reported that the member genes of class I have the function of regulating plant dwarf. The rice homeobox gene OSH15 (Oryza sativa homeobox) is a member of the knotted1-type homeobox gene family. Loss-of-function mutations in the rice homeobox gene OSH15 affect the architecture of internodes resulting in dwarf plants (Sato et al., 1999). The bp mutant of *Arabidopsis* which exhibits bends in inflorescence stems and pedicels as well as reductions in their lengths (Douglas et al., 2002). These data demonstrate a previously unknown link between KNOX gene activity and dicot stem development, whether PbKNOXs can cause plant dwarfing in pear is also unknown, which will be the focus of our next research.

## Conclusion

In this study, we identified 19 PbKNOX TFs from pear based on genome sequences. *PbKNM1* and *PbKNM2* are *KNM* genes identified in pear for the first time. Class I genes play an important role in the function of the meristem and growth and development. Class II genes play an important role in the response to salt stress and drought stress. Analysis of *PbKNOXs* expression in vigorous and dwarf pear trees and hybrid progeny revealed that the expression of class I genes was significantly correlated with height. The results of our study provide candidate genes that could be used to modify the height of pear. Our study also provides new insights that could be used by future studies to clarify the function of *PbKNOX* genes.

## Data Availability Statement

The original contributions presented in the study are included in the article/Supplementary Material, and further inquiries can be directed to the corresponding author.

## Author Contributions

JL, JS, and CL contributed to the conception and design of the study. JL, CZ, JH, XF, and HX organized the database. JL performed the statistical analysis and wrote the first draft of the manuscript. RW, DL, YY, and ZC provided the resources. JL and JS contributed to project administration and funding acquisition. All authors contributed to the article and approved the submitted version.

## Funding

This work was supported by the National Key Research and Development Program of China (2019YFD1001404-3), the Agricultural Improved Variety Project Program of Shandong Province (2019LZGC008), the Natural Science Foundation of Shandong Province (ZR2020QC150), and Open Funds of the State Key Laboratory of Crop Genetics and Germplasm Enhancement (no. ZW202004).

## Conflict of Interest

The authors declare that the research was conducted in the absence of any commercial or financial relationships that could be construed as a potential conflict of interest.

## Publisher’s Note

All claims expressed in this article are solely those of the authors and do not necessarily represent those of their affiliated organizations, or those of the publisher, the editors and the reviewers. Any product that may be evaluated in this article, or claim that may be made by its manufacturer, is not guaranteed or endorsed by the publisher.

## Footnotes

^1^ https://www.rosaceae.org/species/pyrus/all

^2^ https://web.expasy.org/protparam

^3^ http://www.genscript.com/psort/wolf_psort.html

^4^ http://bioinformatics.psb.ugent.be/webtools/plantcare/html/
