# Supplementary material for: Genome-Wide Analysis of KNOX Transcription Factors and Expression Pattern of Dwarf-Related KNOX Genes in Pear
Source: Front Plant Sci. 2022 Jan 28;13:806765. doi: 10.3389/fpls.2022.806765 (PMC8831332; doi:10.3389/fpls.2022.806765)
Supplement: Supplementary file 2 [file Table_2.DOCX]

**Supplementary Table 2. List of qRT-PCR Primers**

| **Primers** | **Forward/Reverse** | **Sequences (5′-3′)** |
| --- | --- | --- |
| Actin | F | CCTTCAATGTGCCTGCTATGTATGT |
|  | R | CCAGCAAGGTCCAGACGAAGAAT |
| PbKNOX6 | F | CTCCTTCATTCTCCACAACCCAG |
|  | R | GCCATGATCTTAGCCTTGACAGT |
| PbKNOX2 | F | ATCATAGCCTGAGCTCCAACGAA |
|  | R | CCTCAAACTCCTGTCTGGCAAC |
| PbKNOX9 | F | AAAGCTAAGATTATCGCTCACC |
|  | R | CTGCTCTAACCTAGCCACC |
| PbKNOX10 | F | CCTTCCTTCTCCACAACCCA |
|  | R | ACATGATCTTAGCCTTGACAGT |
| PbKNOX11 | F | GAGGATTTTCACAGGATGAACGC |
|  | R | GATCATCCACCACATGACCAC |
| PbKNOX12 | F | CATCTAATTTCCAGGAGACCCAC |
|  | R | AATGGCCTCGTAAGATCCG |
| PbKNOX1 | F | GCCCACCCTCAGTACTCTAACCT |
|  | R | TCCCAGAAGACCGCTGTCG |

**Supplementary Table 3. Functional prediction of cis acting elements**

| **Acting elements** | **Description** |
| --- | --- |
| P-box | gibberellin-responsive element |
| CAT-box | cis-acting regulatory element related to meristem expression |
| GCN4_motif | cis-regulatory element involved in endosperm expression |
| ARE | cis-acting regulatory element essential for the anaerobic induction |
| ABRE | cis-acting element involved in the abscisic acid responsiveness |
| RY-element | cis-acting regulatory element involved in seed-specific regulation |
| LTR | cis-acting element involved in low-temperature responsiveness |
| G-box | cis-acting regulatory element involved in light responsiveness |
| TCA-element | cis-acting element involved in salicylic acid responsiveness |
| O2-site | cis-acting regulatory element involved in zein metabolism regulation |
| MBS | MYB binding site involved in drought-inducibility |
| AT-rich sequence | element for maximal elicitor-mediated activation (2copies) |
| MRE | MYB binding site involved in light responsiveness |
| TC-rich repeats | cis-acting element involved in defense and stress responsiveness |
| CGTCA-motif | cis-acting regulatory element involved in the MeJA-responsiveness |
| TGACG-motif | cis-acting regulatory element involved in the MeJA-responsiveness |
| TGA-element | auxin-responsive element |
| CCAAT-box | MYBHv1 binding site |
| GARE-motif | gibberellin-responsive element |
| ACE | cis-acting element involved in light responsiveness |
| AACA_motif | involved in endosperm-specific negative expression |
| GC-motif | enhancer-like element involved in anoxic specific inducibility |
| AuxRR-core | cis-acting regulatory element involved in auxin responsiveness |

**Supplementary Table 4 Ka/Ks analysis for *KNOX* duplicated genes of pear.**

| **Sequence** | **Ka** | **Ks** | **Ka/Ks** |
| --- | --- | --- | --- |
| PbKNM1&PbKNM2 | 0.958271 | 1.17197 | 0.817657 |
| PbKNOX6&PbKNOX10 | 0.996094 | 1.01795 | 0.978528 |
| PbKNOX16&PbKNOX5 | 0.981282 | 1.07836 | 0.909981 |
| PbKNOX1&PbKNOX2 | 0.986931 | 1.05157 | 0.93853 |
| PbKNOX8&PbKNOX14 | 0.961847 | 1.14681 | 0.838712 |
| PbKNOX15&PbKNOX13 | 0.969036 | 1.1251 | 0.861292 |
