# Supplementary figures and images for: Genome-Wide Analysis of KNOX Transcription Factors and Expression Pattern of Dwarf-Related KNOX Genes in Pear
Source: Front Plant Sci. 2022 Jan 28;13:806765. doi: 10.3389/fpls.2022.806765 (PMC8831332; doi:10.3389/fpls.2022.806765)

Figure S1


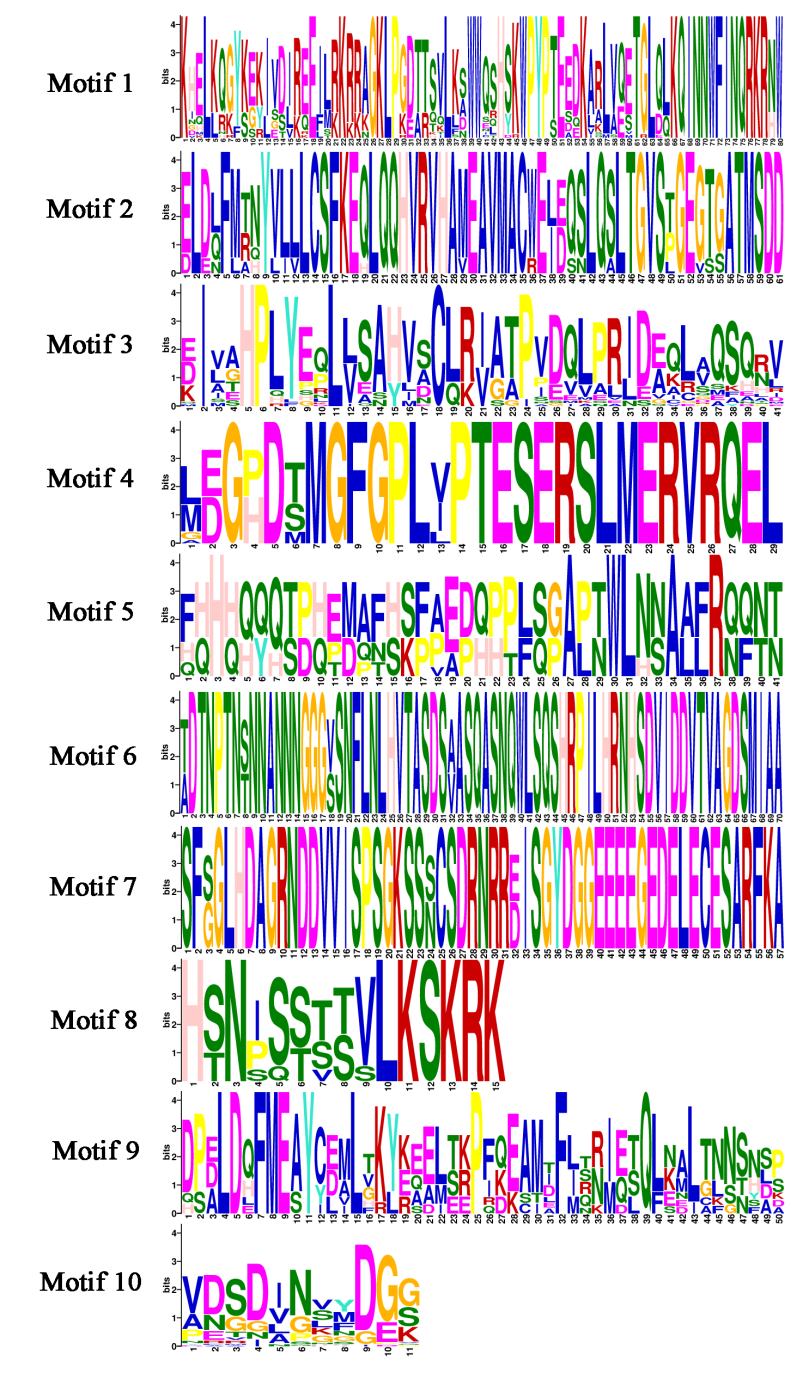


Figure S1 Protein sequences of conserved domains in KNOXs.

Supplement: Supplementary file 3 [file Data_Sheet_1.docx]
